# Antibacterial Activity and Anxiolytic Effect in Adult Zebrafish of Genus *Lippia* L. Species

**DOI:** 10.3390/plants12081675

**Published:** 2023-04-17

**Authors:** Carla de Fatima Alves Nonato, Emerson Vinicius Silva de Melo, Cicera Janaine Camilo, Maria Kueirislene Amâncio Ferreira, Jane Eire Alencar de Meneses, Antonio Wlisses da Silva, Hélcio Silva dos Santos, Jaime Ribeiro-Filho, Joanda Paolla Raimundo e Silva, Josean Fechine Tavares, Irwin Rose Alencar de Menezes, Henrique Douglas Melo Coutinho, Grażyna Kowalska, Tomasz Baj, Radosław Kowalski, José Galberto Martins da Costa

**Affiliations:** 1 Postgraduate Program in Biological Chemistry, Department of Biological Chemistry, Regional University of Cariri, Crato 63105-000, CE, Brazil; 2 Research Laboratory of Natural Products, Department of Biological Chemistry, Regional University of Cariri, Crato 63105-000, CE, Brazil; 3 Postgraduate Program in Biotechnology, Northeast Biotechnology Network, State University of Ceará, Fortaleza 60714-903, CE, Brazil; 4 Postgraduate Program in Natural Sciences, State University of Ceará, Fortaleza 60714-903, CE, Brazil; 5 General Coordination, Oswaldo Cruz Foundation (FIOCRUZ), Eusébio 61773-270, CE, Brazil; 6 Multiuser Laboratory of Characterization and Analysis, Federal University of Paraíba, João Pessoa 58051-900, PB, Brazil; 7 Laboratory of Pharmacology and Molecular Chemistry, Department of Biological Chemistry, Regional University of Cariri, Crato 63105-000, CE, Brazil; 8 Laboratory of Microbiology and Molecular Biology, Department of Biological Chemistry, Regional University of Cariri, Crato 63105-000, CE, Brazil; 9 Department of Tourism and Recreation, University of Life Sciences in Lublin, 15 Akademicka Str., 20-950 Lublin, Poland; 10 Department of Pharmacognosy with Medicinal Plants Garden, Medical University of Lublin, 1 Chodźki Str., 20-093 Lublin, Poland; 11 Department of Analysis and Food Quality Assessment, University of Life Sciences in Lublin, 8 Skromna Str., 20-704 Lublin, Poland

**Keywords:** verbenaceae, essential oil, phenolic compounds, antibacterial, anxiolytic, toxicity, zebrafish

## Abstract

Species belonging to the genus *Lippia* are used worldwide as foods, beverages, and seasonings. Studies have demonstrated that these species have antioxidant, sedative, analgesic, anti-inflammatory, and antipyretic activities. This work aimed to evaluate the antibacterial activity and anxiolytic effect by different pathways of essential oils and ethanolic extracts of three species of *Lippia* (*Lippia alba*, *Lippia sidoides*, and *Lippia gracilis*). The ethanolic extracts were characterized by HPLC-DAD-ESI-MS^n^ and their phenolics were quantified. The antibacterial activity was evaluated by determining the minimal inhibitory concentration and modulation of antibiotic activity, and toxic and anxiolytic effects were evaluated in the zebrafish model. The extracts showed compositions with a low ratio and shared compounds. *L. alba* and *L. gracilis* showed higher amounts of phenols and flavonoids, respectively. All extracts and essential oils presented antibacterial activity, especially those obtained from *L. sidoides*. On the other hand, *L. alba* extract presented the most significant antibiotic-enhancing effect. The samples were not toxic after 96 h of exposure, but showed an anxiolytic effect through modulation of the GABA_A_ receptor, while *L. alba* extract acted via modulation of the 5-HT receptor. This new pharmacological evidence opens horizons for therapeutic approaches targeting anxiolytic and antibacterial therapies and food conservation using these species and their constituents.

## 1. Introduction

Anxiety disorders are among the ten major global causes of disabilities. In 2015, Brazil was considered by the World Health Organization (WHO) as the country with the highest rate of anxiety disorders in the Americas, affecting 9.3% of the population [1]. Benzodiazepines (BZD), which act as positive allosteric modulators of the GABA_A_ receptor, have historically been used as the mainstay of therapy against anxiety for decades, also exhibiting amnesiac, anticonvulsant, sedative, and muscle relaxant effects. However, their chronic use is known to induce tolerance and cause considerable side effects [2,3].

In the list of global health issues, antimicrobial resistance stands out for its worrying spread. Several pathogenic bacteria have developed resistance to first-line antibiotics, causing intractable infections, which, therefore, are associated with high morbidity and mortality rates owing to the lack of effective therapies [4,5].

Importantly, developing new treatment options to combat antibiotic resistance is urgently needed before the inexistence of effective prevention measures and the low availability of antibiotics [6]. In addition, like anxiolytic drugs, antibacterial agents are not free from side effects; therefore, searching for a novel, safe, and effective compound remains an important research topic [7].

Medicinal plants are traditionally used for the treatment of a great variety of diseases. Moreover, many species have served as raw materials for the extraction of bioactive compounds whose health-promoting benefits have long been demonstrated [8]. Studies have shown that different plant extracts and natural products have pharmacological potential, including dietary phytochemicals such as terpenes and phenolic compounds, among others [7].

The species of the genus *Lippia* are used worldwide as food, beverages, and seasonings. Previous research has demonstrated that these species are helpful in treating diseases of the gastrointestinal and respiratory tracts, which is in line with their well-described analgesic, anti-inflammatory, antipyretic [9], antioxidant, antiulcerogenic, antiseptic, and antimicrobial activities. Importantly, studies have demonstrated the effects of *Lippia* species in the central nervous system, including analgesic, anxiolytic, anticonvulsant, sedative, and antinociceptive [10]. On the other hand, the toxic effects of many species in this genus remain to be better investigated.

Considering the evidence that *Lippia* species have the potential to be used in drug development, this study aimed to evaluate the antibacterial and anxiolytic effects of three species of the genus *Lippia* found in the region of Cariri-Ceará, Brazil.

## 2. Results and Discussion

### 2.1. Chemical Composition

The three ethanol extracts were essentially rich in phenolic compounds, as shown in Table 1. *Lippia alba* ethanolic extract (LaEE) showed a greater diversity of compounds, containing 5 iridoids, 1 phenylethanoid glycoside, 1 oxylipin, 1 fatty acid, and 11 flavonoids. While *Lippia sidoides* ethanolic extract (LsEE) and *Lippia gracilis* ethanolic extract (LgEE) presented only flavonoids, with 12 and 15 compounds, respectively, some compounds were identified in more than one sample, such as emodin-8-O-glycoside (20) in LaEE and LsEE; luteolin-6-O-glycoside (16), eriodictyol (23), and luteolin (26) in LsEE and LgEE; and the flavonoids naringenin (24) and cirsimaritin (34) were present in all studied extracts.

Iridoid glycosides are secondary metabolites known to be found in species of the Verbenaceae family [38]. In ethanolic and methanolic extracts from *L. alba* leaves, the presence of geniposidic acid (2) and shanzhiside methyl ester (4) was reported [39,40]. On the other hand, for the compounds monotropein (1), loganic acid (3), and loganin (7), this is the first report for extracts of this species. Commonly, 8-epi-loganin is found in different extracts of *L. alba*, which is a stereoisomer of loganin [39,40,41]. The difference in composition between individuals of the same species may be linked to the variation in abiotic factors to which they are subjected, such as temperature, humidity, and precipitation, among others [42].

Acteoside (12), also called Verbacoside, is a phenylethanoid glycoside already found in extracts of *L. alba* [39,43]. It is recognized for its biological activities, such as an anticonvulsant, antiparkinsonian, cytotoxic, hypotensive, anticancer, antioxidant, and so on [44]. Tuberonic acid glycoside (5), on the other hand, was only seen in extracts of *L. graveolens* Kunth and *L. citriodora* (Palau) Kunth [45,46], with this being the first report in *L. alba*.

The chemical compositions of the ethanolic extract of *Lippia* species showed an abundance of secondary metabolites of the flavonoids class [45], corroborating with the results of this study. The flavonoids apigenin-7-*O*-glucuronide (22), tricin-7-*O*-glucuronide (21), isorhamnetin (27), and naringenin appear in different extracts of *L. alba* [39,43,47], as well as taxifolin (8), luteolin, naringenin, and apigenin (30) in *L. sidoides* [48] and naringenin, quercetin (25), orientin (10), and isoorientin (11) in *L. gracilis* [49]. The diglycosylated form of the flavone chrysoeriol (29) has also been reported for *L. alba* [41].

**Figure 1:**
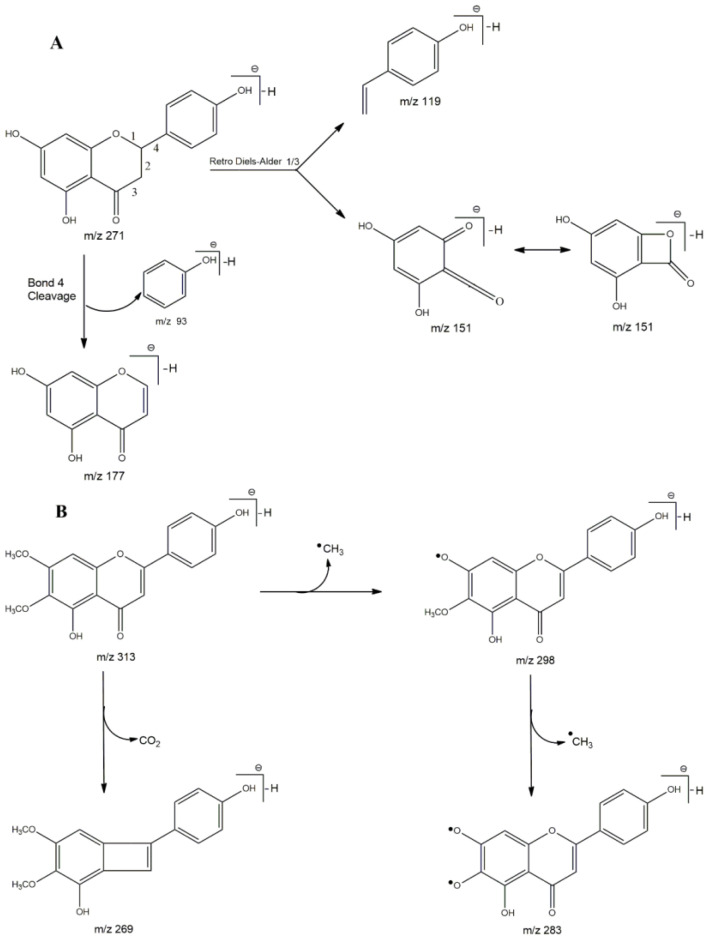
Proposed fragmentation pattern for deprotonated naringenin (**A**) and cirsimaritin (**B**).

Among the flavonoids identified, naringenin and cirsimaritin were found in the three extracts studied, which were reported in the species *L. salviifolia* Cham., *L. velutina* Schauer, *L. balansae* Briq., *L. lupulina* Cham., *L. graveolens* Kunth, *L. citriodora* (Palau) Kunth, *L. javanica* (Burm.f.) Spreng., *L. chevalieri* Moldenke, and *L. lacunosa* Mart. & Schauer [9,45,46,48,50,51]. Thus, we suggest that these flavonoids can be considered as chemical markers for the genus. The use of these markers for medicinal plants is essential considering that bioactivities can, in most cases, be related to a specific chemotype [52]. Figure 1 shows the proposed fragmentations for naringenin and cirsimaritin.

The results show that LaEE has a significantly higher content of total phenolics of 30.11 ± 1.24 mg GA/g Ext., followed by LgEE and LsEE (Table 2). Studies show a high phenolic content for ethanolic, methanolic, and aqueous extracts of *L. alba* leaves, with 117.78 ± 2.69, 367.49 ± 38.90, and 505.11 ± 2.55 µg GAE/g dry weight, respectively [53]. Garmus et al. [54], studying the influence of different types of extraction on the phenolic composition of *L. siodides*, obtained contents ranging between 38.20 ± 0.06 and 230.5 ± 0.1 mg GAE/g extract.

In terms of total flavonoids, LgEE had the highest rate of these compounds, with 8.76 ± 0.27 mg QE/g Ext., followed by LsEE and LaEE (Table 2). However, these contents were lower than those obtained for different extracts of *L. sidoides*, with 43.5 ± 0.3 to 262.3 ± 0.4 mg QE/g extract, and of *L. alba*, with 371.33 ± 4.50 to 463.94 ± 6.49 g QE/gm dry weight [53,54]. Many studies consider the influence of genetic factors, abiotic factors, seasonality, and type of extraction on the composition of a species [55].

The results show that total flavonoid content is equivalent to 93.23%, 88.75%, and 23.21% of the total phenolic values obtained for LsEE, LgEE, and LaEE, respectively. These percentages agree with the compositions identified in the HPLC-DAD-ESI-MS^n^ analysis. Many studies presented in the literature data, with chemical compositions similar to those presented in this manuscript, have reported the significant antioxidant activity of *L. alba*, *L. gracilis*, and *L. sidoides* [43,49,54].

### 2.2. Antibacterial Activity

The present analysis demonstrated that all *Lippia*-derived natural products investigated in this study exhibited significant antibacterial activity (Table 3), with LsEO showing the most remarkable effectiveness in inhibiting bacterial growth, as observed in tests with the strain *S. aureus* Sa 358 (MIC = 53.3 μg/mL). The most potent activity of this extract was observed against most bacterial strains, except for *E. coli* Ec 27, which showed significant sensitivity to all tested samples, as confirmed through the low MICs of the products. We hypothesize that the effectiveness of LsEO may be related to constituents such as thymol and eucalyptol, whose antimicrobial activity has been consistently demonstrated [56,57,58].

**Figure 2:**
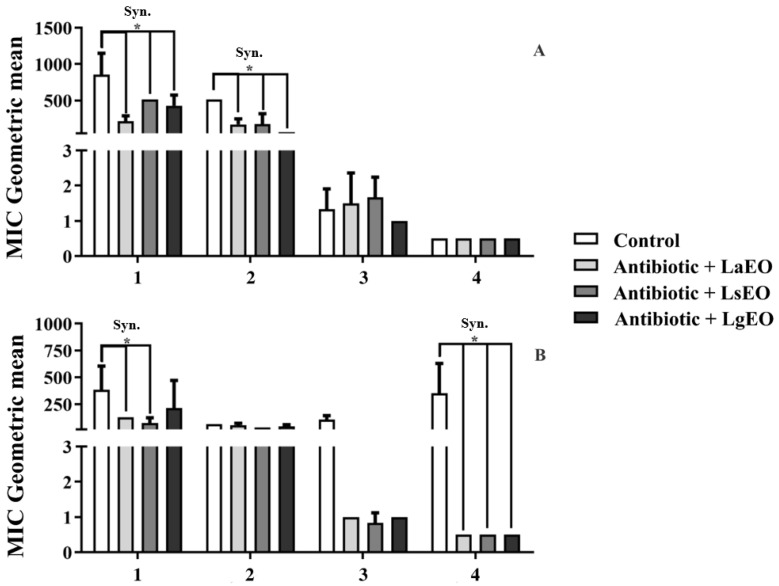
Effects of the essential oils of *L. alba* (LaEO), *L. sidoides* (LsEO), and *L. gracilis* (LgEO) on the antibiotic activity of amikacin (1), gentamicin (2), benzylpenicillin (3), and cephalothin (4) against *Staphylococcus aureus* Sa 358 (**A**) and *Escherichia coli* Ec 27 (**B**). Syn.: synergism; *: *p* < 0.05 (ANOVA and Bonferroni’s post-test).

**Figure 3:**
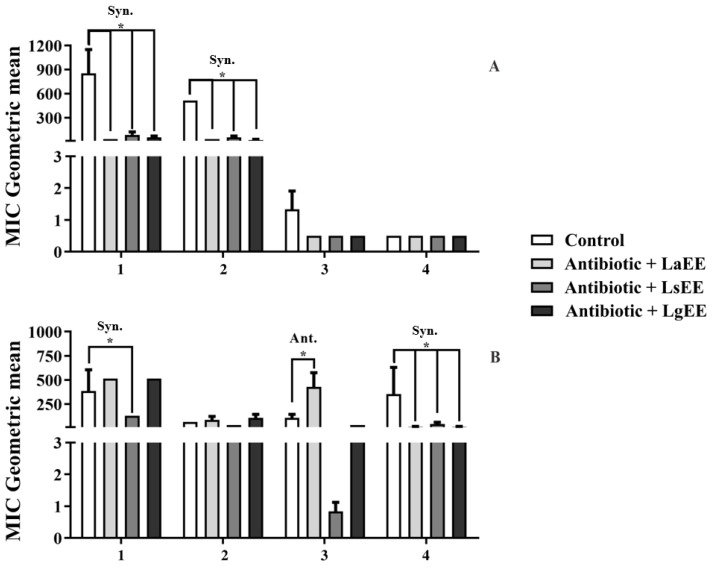
Effects of the ethanolic extracts of *L. alba* (LaEE), *L. sidoides* (LsEE), and *L. gracilis* (LgEE) on the antibiotic activity of amikacin (1), gentamicin (2), benzylpenicillin (3), and cephalothin (4) against *Staphylococcus aureus* Sa 358 (**A**) and *Escherichia coli* Ec 27 (**B**). Syn.: synergism; Ant.: antagonism; *: *p* < 0.05 (ANOVA and Bonferroni’s post-test).

Figure 2 and Figure 3 show the antibacterial activity of conventional antibiotics in association with essential oils and ethanolic extracts of *Lippia* species against *Staphylococcus aureus* Sa 358 and *Escherichia coli* Ec 27. The experiments with the *S. aureus* strain (Figure 2A) revealed that the combination of amikacin with LaEO resulted in a significant synergistic effect as the MIC of the antibiotic was reduced from 853.3 μg/mL to 213.3 μg/mL. A similar effect was observed when this antibiotic was associated with LsEO and LgEO, which reduced its MIC to 512 μg/mL and 426.6 μg/mL, respectively. When associated with LaEO and LsEO, the MIC of gentamicin was reduced from 512 μg/mL to 170.6 μg/mL and 173.3 μg/mL, respectively, whereas for the combination with LgEO, the antibiotic MIC was reduced to 64 μg/mL, indicating a more remarkable antibiotic-enhancing effect.

Previous research has demonstrated that the essential oils of *Lippia* species can potentiate the activity of antibiotics. It was observed that, with the addition of *L. alba* essential oil (at a concentration of 12%), the diameter of the erythromycin-mediated inhibition zone against *S. aureus* ATCC 25923 increased by 221.4% [59]. Moreover, the essential oil of *L. sidoides* significantly reduced the MIC of gentamicin and neomycin against *S. aureus* ATCC 12624 [60]. In addition, studies have demonstrated the effectiveness of essential oils obtained from these species against food-borne pathogens, indicating that they have potential applications in the food industry [61].

Citral initially affects membrane structure, membrane-associated electron transfer, and cellular respiration, leading to rapid energy depletion and, consequently, bacterial cell death [62]. Thymol, on the other hand, acts through cell membrane disruption, biofilm reduction, motility inhibition, inhibition of membrane-bound ATPases, and inhibition of efflux pumps [57]. These mechanisms may be associated with the action of the tested essential oils.

When combined with beta-lactams (Figure 2A), none of the essential oils significantly improved their antibacterial effects. In this context, the most remarkable effect was obtained with LgEO, which reduced the MIC of benzylpenicillin from 1.3 μg/mL to 1 μg/mL. On the other hand, combining this antibiotic with LaEO and LsEO resulted in antagonistic effects, as the MIC increased to 1.5 μg/mL and 1.6 μg/mL, respectively. Finally, none of these essential oils affected the antibacterial effect of cephalothin. This finding is corroborated by the study of Veras et al. [60]. They observed that the essential oil of *L. sidoides* failed in modulating the activity of benzylpenicillin and ceftriaxone against Gram-positive and Gram-negative bacteria.

All combinations between essential oils and antibiotics resulted in synergistic effects in experiments with the *E. coli* strain (Figure 2B). Adding LaEO and LsEO decreased the MIC of amikacin from 384 μg/mL to 128 μg/mL and 74.6 μg/mL, respectively. Interestingly, when combined with cephalothin, each essential oil reduced the antibiotic MIC from 352 μg/mL to 0.5 μg/mL. The combination of LaEO or LgEO decreased the MIC of benzylpenicillin from 106.6 μg/mL to 1 μg/mL. Similarly, an MIC of 0.83 μg/mL resulted from the association of this antibiotic with LsEO. According to the literature, the lipophilic character of essential oils favors their interaction with the lipopolysaccharides of the outer membrane of the cell wall of Gram-negative bacteria, altering its structure and function, which may trigger cell lysis [63].

The ethanolic extracts of the tested species showed significant synergistic effects when combined with aminoglycosides against *S. aureus* (Figure 3A). In this context, the most significant effect was observed with LaEE, whose combination decreased the MIC of amikacin from 853.3 μg/mL to 32 μg/mL. Similarly, LgEE and LsEE decreased the antibiotic MIC to 53.3 μg/mL and 85.3 μg/mL, respectively. Finally, the MIC of gentamicin decreased from 512 μg/mL to 21.3 μg/mL, 32 μg/mL, and 53.3 μg/mL in the presence of LaEE, LgEE, and LsEE, respectively.

Compounds present in ethanolic extracts, such as quercetin, luteolin, pinocebrin, and apigenin, among others, are known for their antibacterial potential [64,65]. It is reasonable to hypothesize that the antibiotic-enhancing effects of these extracts are associated with the presence of flavonoids in their compositions. These secondary metabolites are known to interfere with bacteria growth through mechanisms that involve inhibition of nucleic acid synthesis, changes in the function and permeability of the cytoplasmic membrane, inhibition of energy metabolism, reduction in cell adhesion and biofilm formation of biofilm, and inhibition of porins in the cell membrane [66].

Unlike the experiments with *S. aureus*, the results obtained with *E. coli* cultures (Figure 3B) were quite heterogeneous. While LsEE significantly reduced the MIC of amikacin (from 384 μg/mL to 128 μg/mL), the association with LsEE and LgEE resulted in an antagonistic effect as the antibiotic MIC increased to 512 μg/mL. Moreover, LaEE antagonized the effect of benzylpenicillin, increasing its MIC from 106.6 μg/mL to 426.6 μg/mL. On the other hand, all extracts potentiated the effects of cephalothin. Antagonistic effects from the combination of different antibiotics and natural products are reported in the literature. Evidence indicates that the mechanisms underlying this phenomenon involve competition by the active site and chelation, which frequently results in decreased antibiotic activity [67].

### 2.3. Effects of Lippia Species in the Zebrafish Experimental Model

**Figure 4:**
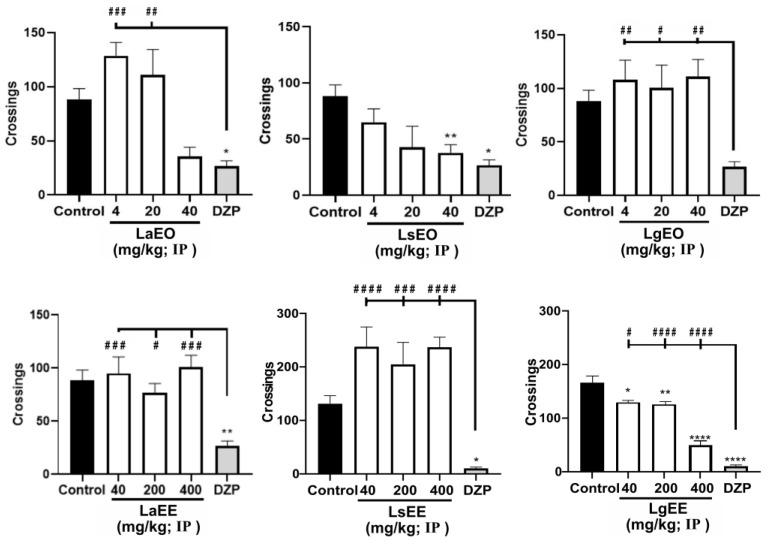
Effect of essential oils and ethanolic extracts of *L. alba*, *L. sidoides*, and *L. gracilis* on the locomotion of adult zebrafish animals in the open field test (0–5 min). The values represent the mean ± standard error of the mean of six animals/group; ANOVA followed by Tukey test (* *p* < 0.05, ** *p* < 0.01, **** *p* < 0.0001, vs. control; # *p* < 0.05, ## *p* < 0.01, ### *p* < 0.001, #### *p* < 0.0001 vs. DZP). LaEO: *Lippia alba* essential oil; LsEO: *Lippia sidoides* essential oil; LgEO: *Lippia gracilis* essential oil; LaEE: *Lippia alba* ethanolic extract; LsEE: *Lippia sidoides* ethanolic extract; LgEE: *Lippia gracilis* ethanolic extract; DZP: diazepam.

As shown in Table 4, none of the extracts and essential oils induced significant toxicity to adult zebrafish after 96 h (acute toxicity), as there was no death or apparent anatomical alteration in the animals during this period. In the open field test (Figure 4), LgEO and LaEO (at the lowest dose) did not cause motor impairment in the animals. On the other hand, like DZP, the higher doses of LaEO and LsEO altered locomotion. Regarding the ethanolic extracts, LsEE, even at the highest dose, did not affect the locomotion of the animals, different from what was observed following the treatment with LgEE.

Anxiolytic drugs such as BZD are known to reduce the locomotion of zebrafish animals, providing a model to analyze the impact of chemical substances on the central nervous system (CNS) [68,69]. This study showed that, although the natural products caused no evident toxicity, some of them affected the locomotion of the animals, indicating CNS action. Ethnobotanical reports have indicated the use of medicinal plants to treat CNS disorders such as depression, epilepsy, anxiety, and pain. However, the effectiveness of most of these species remains to be confirmed experimentally [10].

**Figure 5:**
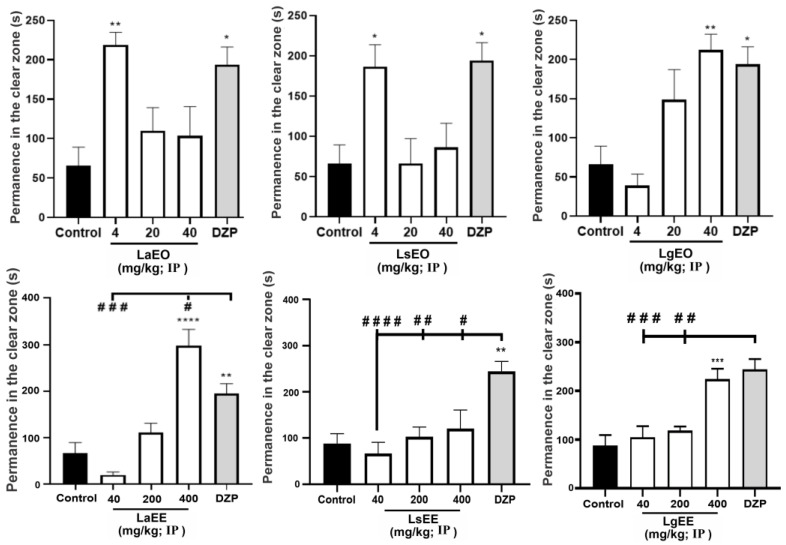
The anxiolytic effect of essential oils and ethanolic extracts of *L. alba*, *L. sidoides*, and *L. gracilis* in the light/dark test (0–5 min). The values represent the mean ± standard error of mean of six animals/group; ANOVA followed by Tukey’s test (* *p* < 0.05, ** *p* < 0.01, *** *p* < 0.001, **** *p* < 0.0001, vs. control; # *p* < 0.05, ## *p* < 0.01, ### *p* < 0.001, #### *p* < 0.0001 vs. DZP). LaEO: *Lippia alba* essential oil; LsEO: *Lippia sidoides* essential oil; LgEO: *Lippia gracilis* essential oil; LaEE: *Lippia alba* ethanolic extract; LsEE: *Lippia sidoides* ethanolic extract; LgEE: *Lippia gracilis* ethanolic extract; DZP: diazepam.

The light/dark test follows the observation that experimental animals escape to the dark compartment under anxiogenic conditions as an adaptive natural antipredatory response. Thus, studies in adult zebrafish animals have shown that anxiolytic compounds reduce their preference for the dark compartment [70]. The treatment with LgEO, LaEE, LaEO, LsEO, and LgEE induced an anxiolytic behavior that increased the permanence of the animals in the light area of the aquarium (Figure 5), similar to the DZP group. However, the treatment with LsEE did not affect the anxiety behavior of the animals. Previous research has demonstrated the sedative, anxiolytic, and anticonvulsant effects of *L. alba* [10] and *L. sidoides* [71], probably owing to the presence of constituents such as citral, β-myrcene, limonene, thymol, and carvacrol [72,73]. Moreover, naringenin and quercetin, commonly found in *Lippia* species, demonstrated anxiolytic activity in the zebrafish model [74,75]. In addition, other species of the Verbenaceae family demonstrated anxiolytic potential in the same model, corroborating the present findings [76].

**Figure 6:**
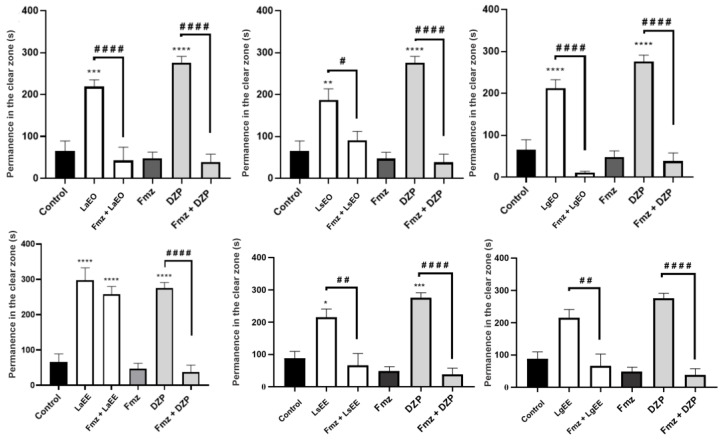
Involvement of GABAergic neurotransmission in the anxiolytic effect of essential oils and ethanolic extracts of *L. alba*, *L. sidoides*, and *L. gracilis* in the light/dark test (0–5 min). The values represent the mean ± standard error of the mean of six animals/group; ANOVA followed by Tukey’s test (* *p* < 0.05, ** *p* < 0.01, *** *p* < 0.001, **** *p* < 0.0001, vs. control; # *p* < 0.05, ## *p* < 0.01, #### *p* < 0.0001 vs. DZP or *Lippia*). LaEO: *Lippia alba* essential oil; LsEO: *Lippia sidoides* essential oil; LgEO: *Lippia gracilis* essential oil; LaEE: *Lippia alba* ethanolic extract; LsEE: *Lippia sidoides* ethanolic extract; LgEE: *Lippia gracilis* ethanolic extract; DZP: diazepam; Fmz: flumazenil.

The involvement of GABAergic neurotransmission in the anxiolytic effect of the natural products was evaluated through pre-treatment with the GABA_A_ receptor antagonist flumazenil. It was observed that this treatment blocked the anxiolytic effect of all samples except LaEE (Figure 6), increasing the animals’ permanence in the aquarium’s dark region. This finding suggests that the tested natural products have constituents capable of activating the GABA_A_ receptor in the same region as benzodiazepines, such as DZP [77]. This hypothesis is corroborated by studies demonstrating the effectiveness of *L. alba* essential oils as anxiolytics and anesthetics through interference with GABAergic neurotransmission in zebrafish and silver catfish models [78,79]. Flavonoids such as quercetin, apigenin, luteolin, vitexin, isovitexin, naringenin, and eriodctyol act as second-order modulators of first-order modulation by BZD and modify the Fmz-insensitive modulation of the GABAA receptor, where incorporation of electronegative groups into C-6 and C-3’ in the flavone backbone increases the affinity for the BZD binding site [80].

**Figure 7:**
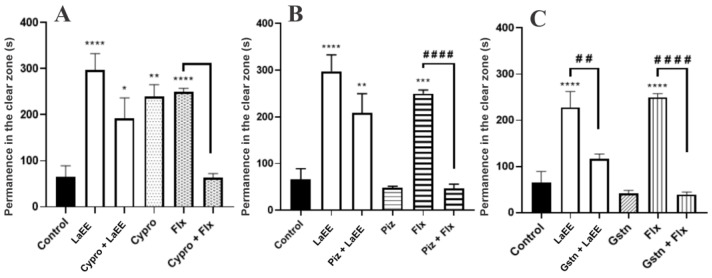
Effects of the pre-treatment with cyproheptadine (**A**), pizotifen (**B**), and granisetron (**C**) in the anxiolytic effect of *L. alba* ethanolic extract through the light/dark test (0–5 min). The values represent the mean ± standard error of the mean of six animals/group; two-way ANOVA followed by Tukey’s test (* *p* < 0.05, ** *p* < 0.01, *** *p* < 0.001, **** *p* < 0.0001, vs. control; ## *p* < 0.01, #### *p* < 0.0001 vs. Flx or *Lippia*). LaEE: *Lippia alba* ethanolic extract; Cypro: cyproheptadine; Piz: pizotifen; Gstn: granisetron; Flx: fluoxetine.

As LaEE treatment showed no interference with GABAergic neurotransmission, we investigated whether this extract could exert its anxiolytic effects through interference with the serotonergic neurotransmission, which was investigated using 5-HT receptor antagonists. Pre-treatment with granisetron blocked the anxiolytic effect of LaEE (Figure 7), while pizotifen and cyproheptadine treatments did not affect the activity of the extract, indicating that LaEE has anxiolytic effects associated with the activation of 5-HT_3A/3B_ receptors.

It has been demonstrated that neurotransmitters, such as serotonin (5-HT), dopamine, and gamma-aminobutyric acid (GABA), can regulate the activation of neurons that govern behaviors and emotions [81,82]. At the same time, high levels of 5-HT can induce anxiety-like effects, while low levels of 5-HT cause anxiolytic behavior in the zebrafish model [83]. 5-HTR_3_ is located in the regions of the brain that regulate mood and emotions; therefore, its activation may be associated with the anxiolytic and antidepressant effects of drugs [84]. Accordingly, the aqueous extract of *L. multiflora* Moldenke, whose composition is characterized by the high content of flavonoids, showed an anxiolytic effect mediated by the activation of serotonergic receptors such as 5-HT_2C/2B_, 5-HT_1A_, and 5-HT_2A/2C_ [85]. In silico and in vivo analyses show the antidepressant and anxiolytic effect of apigenin, a flavonoid present in LaEE, through its effective coupling to serotonin receptors, possibly acting through 5-HT_2A_ inhibition and 5-HT_1A_ agonism [86].

## 3. Materials and Methods

### 3.1. Botanical Material and Extract Preparation

The collection of the botanical material as well as the extraction and composition of the essential oils used in this study are described in Nonato et al. [56]. Briefly, the leaves of *Lippia alba* (Mill.) N.E.Br. ex Britton & P.Wilson and *Lippia sidoides* Cham. were collected in the botanical garden of the Regional University of Cariri (7°14′20.1″ S 39°24′53.1″ W) in April 2019 and the voucher specimen of each species was registered in the Herbarium Caririense Dárdano de Andrade Lima (HDCAL/URCA) under identification numbers 13,907 and 3038, respectively. The leaves of *Lippia gracilis* Schauer were collected in the municipality of Crato, Ceará, Brazil (7°13′05.2″ S 39°25′44.9″ W) in April 2019, and its voucher specimen was registered in the Herbarium Prisco Bezerra of the Federal University of Ceará, under registration number 44,456.

The essential oils were extracted by hydrodistillation in a Clevenger-type apparatus. Briefly, fresh leaves were crushed and extracted for 2 h. After extraction, the essential oils of *L. alba* (LaEO), *L. sidoides* (LsEO), and *L. gracilis* (LgEO) were dried with Na_2_SO_4_ [56]. In another set of extractions, fresh leaves of these species (500 g of each) were subjected to exhaustive maceration in 99% ethanol for 72 h. The solutions obtained were concentrated in an evaporator at 50 °C under reduced pressure, obtaining a yield of 31.6%, 11.5%, and 1.48% for the ethanolic extracts of *L. alba* (LaEE), *L. sidoides* (LsEE), and *L. gracilis* (LgEE), respectively.

### 3.2. Drugs and Reagents

The following substances were used in this study: methanol (MeOH), Na_2_CO_3_, AlCl_3_, CH_3_CO_2_K, gentamycin, amikacin, benzylpenicillin, cephalothin (Sigma Chemical Corporation, San Luis, Missouri, USA); diazepam (DZP; Neo Química^®^), flumazenil (Fmz; Sandoz^®^), dimethylsulfoxide (DMSO; Dynamic^®^), granisetron chloride (Gstn; Corepharma/Middlesex, NJ, USA), pizotifen maleate (Piz; Central Pharmacy of Manipulation/São Paulo, SP, Brazil), fluoxetine (Flx; Eli Lilly/Indianapolis, IN, USA), and cyproheptadine (Cypro; Evidence Pharmaceutical Solutions/Fortaleza, CE, Brazil).

### 3.3. Analysis of Non-Volatile Compounds by HPLC-DAD-ESI-MS^n^

The three extracts were analyzed by Shimadzu HPLC, using a C18 analytical chromatographic column (Kromasil—250 mm × 4.6 mm × 5 μm), coupled to a mass spectrometer (Ion-Trap AmazonX, Bruker or microTOFII, Bruker, Billerica, MA, USA), with ionization by electrospray (ESI). The samples were solubilized in MeOH (1 mg/mL), with subsequent filtration through PVDF (polyvinylidene fluoride) filters, with a mesh size of 0.5 μm. The method used chromatographic-grade MeOH (solvent B) and type I ultrapure H_2_O (Milli-Q), acidified with formic acid (0.1%, *v*/*v*) (solvent A), in a concentration gradient (5 to 100% of B in 95 min). The injection volume was 10 μL and the flow rate was 0.6 mL/min. In the mass spectrometer, the samples were submitted to a sequential fragmentation in MS3. The acquisition parameters in the ion trap and TOF were capillary of 4.5 kV, final plate offset of 500 V, nebulizer gas at 35 psi, dry gas (N_2_) with flow of 8 mL/min, and a temperature of 300 °C. The sample was analyzed in negative ionization mode and the identification of compounds was based on data (MS/MS) reported in the literature.

### 3.4. Quantification of Total Phenols

The quantification of total phenols followed the Folin–Ciocalteu oxidation method proposed by Singleton et al. [87]. Here, 25 µL aliquots of extract concentrations (50 to 1000 µg/mL in MeOH) were added to Folin–Ciocalteu reagent (625 µL, 10%) and Na_2_CO_3_ (500 µL, 7.5%). Absorbances were measured in a spectrophotometer (Kasuaki DR-200BS, Wuxi Hiwell Diatek Instruments, 209 China) at 765 nm after incubation for 15 min at 45 °C and protected from light. For the calibration curve, gallic acid was used as a standard and, for the blank test, MeOH was used. The analysis was performed in triplicate and the results were expressed as mg equivalent of gallic acid per g of extract (mg GA/g Ext.).

### 3.5. Quantification of Total Flavonoids

The total flavonoid content was measured by the AlCl_3_ colorimetric method according to Kosalec et al. [88]. Here, 1160 µL aliquots of extract concentrations (50 to 1000 µg/mL in MeOH) were added to MeOH (760 µL), AlCl_3_ (40 µL, 10%) and CH_3_CO_2_K (40 µL, 0.1 M). Absorbances were measured in a spectrophotometer at 415 nm after incubation for 30 min in the dark. Quercetin was used as a standard for the calibration curve and MeOH was used for the blank test. All analyses were performed in triplicate and the results were expressed as mg quercetin equivalent per g of extract (mg QE/g Ext.).

### 3.6. Antibacterial Activity Analysis

#### 3.6.1. Minimum Inhibitory Concentration Determination

The antibacterial activity was analyzed through the microdilution method according to the CLSI M100 document [89], using the following bacterial strains: *Staphylococcus aureus* Sa 358, *Streptococcus mutans* INCQS 00446, *Escherichia coli* Ec 27, and *Pseudomonas aeruginosa* ATCC 15442.

To this end, the samples were previously diluted to 1024 μg/mL in sterile distilled water and DMSO. The wells on a 96-well plate were filled with a bacterial suspension (10^5^ CFU/mL in 10% BHI medium), and then the samples were added to the wells where serial dilutions were performed to reach concentrations of the natural products ranging from 512 to 8 μg/mL. The plate was incubated at 35 ± 2 °C for 24 h. The reading was performed by colorimetry after adding 25 μL of resazurin solution (0.01%) to each well after incubation. The MIC was defined as the lowest concentration of extracts capable of inhibiting the growth of microorganisms. All tests were performed in triplicate.

#### 3.6.2. Evaluation of Antibiotic-Enhancing Activity by Direct Contact

This study followed the methodology proposed by Coutinho et al. [90] to analyze the effectiveness of essential oils and ethanolic extracts as potentiators of the antibacterial activity of aminoglycosides (amikacin and gentamicin) and beta-lactams (benzylpenicillin and cephalothin) against the multidrug-resistant bacterial strains *Staphylococcus aureus* Sa 358 and *Escherichia coli* Ec 27 (Table 5). To this end, the antibiotics were tested alone or combined with natural products added to the wells at a concentration equivalent to their MIC/8. The plates were incubated and the antibiotic MIC was determined as previously described.

### 3.7. Zebrafish Experimental Model

#### 3.7.1. Animals

Wild-type adult zebrafish animals (*Danio renio*) of both sexes aged between 60 and 90 days (0.4 ± 0.1 g) were obtained from a commercial supplier (Fortaleza, CE). The animals were kept in a glass aquarium containing anti-chlorine-treated water (n = 5/L), at a temperature of 25 ± 2 °C, in light/dark cycles for 24 h. The experiments followed the Ethical Principles of Animal Experimentation and were approved by the Ethics Committee for the Use of Animals (CEUA) of the State University of Ceará (04983945/2021). After the experiments, the animals were euthanized by freezing through immersion in cold water (2–4 °C) until the loss of opercular movements (approximately 10 min).

#### 3.7.2. Acute Toxicity Determination

The acute toxicity evaluation in adult zebrafish animals was conducted according to the Organization of Economic Cooperation and Standard Method of Development [92]. The animals (n = 6/group) were treated intraperitoneally (IP) with 20 μL of essential oils (4, 20, or 40 mg/kg), extracts (40; 200, and 400 mg/kg), or vehicle (3% DMSO). After 96 h, the number of dead animals in each group was counted, and the LD_50_ was determined using the trimmed Spearman–Karber mathematical method with a 95% confidence interval [93].

#### 3.7.3. Evaluation of Locomotor Activity (Open Field Test)

The open field test evaluated changes in the motor system due to sedation or muscle relaxation [94]. The animals (n = 6/group) were treated as previously described, except for a diazepam-treated (4 or 10 mg/kg) group of animals, which was included as a pharmacological control group. After 30 min of the treatments, the animals were placed on water-filled Petri dishes (10 × 15 cm) marked with four quadrants. The locomotor activity was analyzed by counting the line-crossing (LC) frequency.

#### 3.7.4. Anxiolytic Activity Analysis (Light/Dark Test)

The light/dark test can detect the anxiety-like behavior of zebrafish animals because, like rodents, they naturally avoid illuminated areas [84]. A glass aquarium (30 cm × 15 cm × 20 cm), divided into light and dark areas, was filled with up to 3 cm of tap water free of chlorine and heavy metals, which simulated a shallow environment that, unlike the conventional aquarium, is capable of inducing anxiety-like behavior. The animals were treated following the same treatment conditions described in the previous experimental section and, 1 h later, placed individually in the light zone of the aquarium. The anxiolytic effect was measured based on the time spent in the light area of the aquarium within a 5 min time interval of observation [95].

#### 3.7.5. Evaluation of GABAergic Neuromodulation

The animals were pre-treated with Flumazenil (Fmz), a GABA_A_ receptor antagonist, to analyze the interference of extracts and the essential oils on the GABAergic neuromodulation, and thus elucidate the mechanisms underlying the anxiolytic effects of the extracts and essential oils [96]. Briefly, zebrafish (n = 6/group) were pre-treated with Fmz (4 mg/kg) 15 min before the treatment with the natural products. Here, the best dose of each substance was selected according to the light/dark test (4 mg/kg for LaEO and LsEO; 40 mg/kg for LgEO; 400 mg/kg for all extracts). Groups of mice treated with the vehicle (3% DMSO) and DZP (4 mg/kg) were used as negative and pharmacological controls, respectively. Following 30 min of the treatment, the light/dark test was applied as described in the previous section. All treatments were performed intraperitoneally in a volume of 20 μL.

#### 3.7.6. Evaluation of Serotonergic Neuromodulation

The potential participation of the serotoninergic pathway in the anxiolytic effect of LaEE was investigated using the following receptor antagonists: cyproheptadine (5-HT_R2A_ antagonist), pizotifen (5-HT_R1_/5-HT_R2A_/5-HT_R2C_ antagonist), and granisetron (5-HT_R3A_/5-HT_R3B_ antagonist) [96]. Zebrafish animals (n = 6/group) were pretreated orally (p.o) with cyproheptadine (32 mg/kg), pizotifen (32 mg/kg), or granisetron (20 mg/kg). After 15 min, these animals were treated intraperitoneally with LaEE (400 mg/kg), following the anxiolytic effect observed in the light/dark test. The mice treated intraperitoneally with the vehicle (3% DMSO) and fluoxetine (0.05 mg/kg) were used as negative and pharmacological controls, respectively. Following 1 h of the treatment, the light/dark test was applied as described in the previous section.

### 3.8. Statistical Analysis

The data obtained in the antibacterial activity analysis had the normal distribution evaluated and were then analyzed by one-way ANOVA with Bonferroni’s post-test. For the zebrafish tests, the results were expressed as the mean ± standard error of the mean for each group of 6 animals. After confirming the normality of the distribution and homogeneity of the data, the differences between the groups were analyzed by one-way ANOVA (or two-way ANOVA for GABAergic and serotonergic evaluation), followed by Tukey’s test. GraphPad Prism software version 8.0 (GraphPad Software, San Diego, CA, USA) was used in all analyses, and the results with *p* < 0.05 were considered statistically significant.

## 4. Conclusions

According to the results obtained, the ethanolic extracts were essentially rich in flavonoids, with naringenin and cirsimaritin present in the three extracts, indicating the possibility of a chemical marker for this genus. The essential oils and extracts studied showed significant antibacterial and antibiotic effects, demonstrating the ability both to inhibit bacterial growth (especially *L. sidoides*) and to potentiate the activity of conventional antibiotics (especially *L. alba*). Furthermore, these natural products caused anxiolytic behavior in zebrafish through interference in GABAergic and serotoninergic pathways. These showed no toxic effect, demonstrating the safe use of these species both for new biological studies and for ethnopharmacological uses.

The volatile and non-volatile compounds identified in these samples are reported in the literature with the studied biological effects, which strengthens the results obtained here. New studies are needed to verify the correlation of the composition with these potentials, as well as their individual mechanisms of action in silico and in vivo. It is noteworthy that this work is the first report of the activity of all extracts and essential oils of *L. sidoides* and *L. gracilis* in the zebrafish model. This new pharmacological evidence opens horizons for therapeutic approaches targeting anxiolytic and antibacterial therapies, as well as food preservation using these species and their constituents.

## Figures and Tables

**Table 1:** Compounds identified by HPLC-DAD-ESI-MS^n^ in ethanolic extracts of *L. alba*, *L. sidoides,* and *L. gracilis*.

N°	R.T.	[M-H]^−^	Molecular Formula	Error (ppm)	MS^n^ m/z	Assignment	Extract	Reference
1	11.1	389.1083	C_16_H_21_O_11_	1.6	MS^2^ [389.0]: 227.0, 191.0, 165.0, 147.0	monotropein	LaEE	[11]
2	22.3	373.1138	C_16_H_21_O_10_	0.7	MS^2^ [373.0]: 211.0, 193.0, 167.0, 149.0, 123.0	geniposidic acid	LaEE	[12]
3	27.4	375.1294	C_16_H_23_O_10_	0.8	MS^2^ 411.0 [M + Cl−H]^−^: 375.0 MS^3^ [411.0 → 375.0]: 213.0, 169.0, 151.0	loganic acid	LaEE	[13]
4	31.4	405.1406	C_17_H_25_O_11_	0.9	MS^2^ 451.0 [M + HCOOH−H]^−^: 405.0 MS^3^ [451.0 → 405.0]: 373.0, 243.0 MS^4^ [405.0 → 243.0]: 225.0, 123.0, 101.0	shanzhiside methyl ester	LaEE	[12]
5	36.2	387.1656	C_18_H_27_O_9_	1.2	MS^2^ [387.0]: 207.0, 163.0	tuberonic acid glucoside	LaEE	[14]
6	38.8	593.0112	C_27_H_29_O_16_	0.3	MS^2^ [593.0]: 575.0, 503.0, 473.0, 383.0, 353.0	apigenin-6,8-*C*-diglucoside	LgEE	[15]
7	39.5	389.1452	C_17_H_25_O_10_	0.4	MS^2^ 435.0[M + HCOOH−H]^−^: 389.0 MS^3^ [435.0 → 389.0]: 227.0, 101.0	loganin	LaEE	[16]
8	43.2	303.0519	C_15_H_12_O_7_	1.6	MS^2^ [303.0]: 284.9, 176.9, 124.8	taxifolin	LsEE LgEE	[17]
9	43.4	449.1093	C_21_H_22_O_11_	0.5	MS^2^ [449.0]: 286.9 MS^3^ [449.0 → 286.9]: 150.9 MS^4^ [449.0 → 286.9 → 150.9]: 106.9	eriodictyol-7-*O*-glicoside	LsEE	[18]
10	43.4	447.0421	C_21_H_19_O_11_	0.2	MS^2^ [447.0]: 392.9, 356.9, 327.0	orientin	LgEE	[19]
11	44.1	447.0235	C_21_H_19_O_11_	0.7	MS^2^ [447.0]: 429.0, 411.0, 357.0, 327.0	isoorientin	LgEE	[19]
12	44.3	623.1989	C_29_H_35_O_15_	1.2	MS^2^ [623.0]: 461.0, 315.0	acteoside	LaEE	[20]
13	45.8	431.0431	C_21_H_20_O_10_	1.0	MS^2^ [431.0]: 310.9, 340.9 MS^3^ [431.0 → 310.9]: 282.9	vitexin	LgEE	[21]
14	47.2	451.1231	C_21_H_24_O_11_	−2.6	MS^2^ [451.0]: 288.9 MS^3^ [451.0 → 288.9]: 270.9, 166.8, 124.9	3-hydroxyphlorizin	LsEE	[22]
15	48.2	431.0169	C_21_H_20_O_10_	0.9	MS^2^ [431.0]: 413.0, 395.0; 310.9, 341.0 MS^3^ [431.0 → 310.9]: 282.9	isovitexin	LgEE	[21]
16	48.5	447.0940	C_21_H_20_O_11_	−1.3	MS^2^ [447.0]: 284.9 MS^3^ [447.0 → 284.9]: 240.9, 198.8, 174.9, 150.8, 132.9	luteolin-6-*O*-glicoside	LsEE LgEE	[18]
17	49.5	286.9134	C_15_H_12_O_6_	0.4	MS^2^ [286.9]: 269.0, 258.9, 243.0, 201.0, 124.9	dihydrokaempferol	LgEE	[23]
18	50.3	507.1145	C_23_H_23_O_13_	0.1	MS^2^ [507.0]: 345.0, 330.0, 315.0	quercetagetin-dimethyl-*O*-hexoside	LaEE	[24]
19	51.9	435.1296	C_21_H_24_O_10_	0.1	MS^2^ [435.0]: 272.9 MS^3^ [435.0 → 272.9]: 166.8	phloridzin	LsEE	[25]
20	52.5	431.0994	C_21_H_20_O_11_	−2.4	MS^2^ [431.0]: 269.0 MS^3^ [431.0 → 269.0]: 225.0	emodin-8-*O*-glicoside	LaEE LsEE	[26]
21	53.2	505.1002	C_23_H_21_O_13_	2.8	MS^2^ [505.0]: 329.0, 314.0, 299.0	tricin-7-*O*-glucuronide	LaEE	[27]
22	53.4	445.0764	C_21_H_17_O_11_	2.8	MS^2^ [445.0]: 269.0, 175.0 MS^3^ [445.0 → 269.0]: 225.0, 183.0	apigenin-7-*O*-glucuronide	LaEE	[28]
23	55.5	287.0596	C_15_H_12_O_6_	−2.0	MS^2^ [287.0]: 268.8, 150.8, 124.9, 106.9	eriodictyol	LsEE LgEE	[29]
24	60.6	271.0619	C_15_H_11_O_5_	2.7	MS^2^ [270.9]: 176.8, 150.8, 118.9	naringenin	LaEE LsEE LgEE	[29]
25	61.4	300.9852	C_15_H_10_O_7_	0.1	MS^2^ [300.9]: 272.9, 178.8, 150.8	quercetin	LgEE	[30]
26	62.3	285.0408	C_15_H_10_O_6_	−1.3	MS^2^ [284.9]: 266.9, 256.8, 242.9, 240.9, 216.9, 198.9, 174.9, 150.9, 132.9	luteolin	LsEE LgEE	[29]
27	62.9	315.0502	C_16_H_11_O_7_	2.7	MS^2^ [315.0]: 300.0	isorhamnetin	LaEE	[31]
28	64.1	593.1489	C_27_H_30_O_15_	−2.5	MS^2^ [593.0]: 446.9, 284.9 MS^3^ [593.0 → 284.9]: 240.8, 198.7, 174.8, 150.9, 132.9	luteolin-7-*O*-rutinose	LsEE	[32]
29	66.7	299.0555	C_16_H_11_O_6_	2.1	MS^2^ [299.0]: 284.0 MS^3^ [299.0 → 284.0]: 256.0, 227.0, 212.0	chrysoeriol	LaEE	[33]
30	66.9	268.0459	C_15_H_10_O_5_	−1.5	MS^2^ [268.9]: 224.8, 226.9, 200.9, 150.9, 148.8	apigenin	LsEE	[34]
31	67.0	329.0662	C_17_H_13_O_7_	1.6	MS^2^ [329.0]: 314.0 MS^3^ [329.0 → 314.0]: 299.0, 285.0	tricin	LaEE	[33]
32	69.1	373.0925	C_19_H_17_O_8_	1.1	MS^2^ [373.0]: 358.0, 343.0 MS^3^ [373.0 → 358.0]: 343.0 MS^4^ [373.0 → 358.0 → 343.0]: 328.0, 300.0	dihydroxy-tetramethoxy flavone	LaEE	[24]
33	69.5	327.2166	C_18_H_31_O_5_	3.4	MS^2^ [327.0]: 309.0, 291.0, 229.0, 211.0, 209.0, 171.0	oxo-dihydroxy-octadecenoic acid	LaEE	[28]
34	70.0	313.0706	C_17_H_13_O_6_	3.6	MS^2^ [313.0]: 297.9, 283.0, 269.0	cirsimaritin	LaEE LsEE LgEE	[35]
35	71.7	284.9821	C_16_H_14_O_5_	0.6	MS^2^ [284.9]: 190.8, 164.9, 118.9	sakuranetin	LgEE	[36]
36	71.8	343.0823	C_18_H_17_O_7_	0.0	MS^2^ [343.0]: 328.0, 313.0 MS^3^ [343.0 → 328.0]: 313.0, 298.0, 285.0, 270.0	dihydroxy-trimethoxyflavone	LaEE	[24]
37	72.4	254.9103	C_15_H_12_O_4_	0.1	MS^2^ [254.9]: 212.9, 186.9, 150.8, 144.8, 135.8, 124.9	pinocembrin	LgEE	[37]

R.T.: retention time. LaEE: *Lippia alba* ethanolic extract; LsEE: *Lippia sidoides* ethanolic extract; LgEE: *Lippia gracilis* ethanolic extract.

**Table 2:** Total content of phenols and flavonoids in the ethanolic extracts of *L. alba*, *L. sidoides*, and *L. gracilis*.

Samples	Total Phenolics (mg GA/g Ext.)	Total Flavonoids (mg QE/g Ext.)
LaEE	30.11 ± 1.24 a	6.99 ± 0.28 a
LsEE	9.17 ± 0.26 b	8.55 ± 0.10 b
LgEE	9.87 ± 0.71 b	8.76 ± 0.27 b

These results are expressed as mean ± SD (n = 3). Means followed by different letters differ by Tukey’s test with *p* < 0.05. LaEE: *Lippia alba* ethanolic extract; LsEE: *Lippia sidoides* ethanolic extract; LgEE: *Lippia gracilis* ethanolic extract.

**Table 3:** Minimum inhibitory concentrations of essential oils and ethanolic extracts obtained from *L. alba*, *L. sidoides*, and *L. gracilis*.

Bacterial Strains	MIC (µg/mL)					
	LaEO	LsEO	LgEO	LaEE	LsEE	LgEE
*Staphylococcus aureus* Sa 358	256	53.3	512	853.3	128	512
*Streptococcus mutans* INCQS 00446	213.3	106.6	512	≥1024	170.6	853.3
*Escherichia coli* Ec 27	106.6	106.6	426.6	768	74.6	256
*Pseudomonas aeruginosa* ATCC 15442	213.3	128	512	≥1024	298.6	682.6

INCQS: National Institute for Quality Control in Health; ATCC: American Type Culture Collection. LaEO: *Lippia alba* essential oil; LsEO: *Lippia sidoides* essential oil; LgEO: *Lippia gracilis* essential oil; LaEE: *Lippia alba* ethanolic extract; LsEE: *Lippia sidoides* ethanolic extract; LgEE: *Lippia gracilis* ethanolic extract.

**Table 4:** Zebrafish mortality following the treatment with *L. alba*, *L. sidoides*, and *L. gracilis*.

Sample	Mortality				96 h LD_50_ (mg/kg)/CI
	NC	D1	D2	D3	
LaEO	0	0	0	0	>40
LsEO	0	0	0	0	>40
LgEO	0	0	0	0	>40
LaEE	0	0	0	0	>400
LsEE	0	0	0	0	>400
LgEE	0	0	0	0	>400

NC: negative control group (DMSO 3%); D1: dose 1 (4 mg/kg¹; 40 mg/kg²); D2: dose 2 (20 mg/kg¹; 200 mg/kg²); D3: dose 3 (40 mg/kg¹; 400 mg/kg²). LD_50_: half-maximal lethal dose; CI: confidence interval; LaEO: *Lippia alba* essential oil; LsEO: *Lippia sidoides* essential oil; LgEO: *Lippia gracilis* essential oil; LaEE: *Lippia alba* ethanolic extract; LsEE: *Lippia sidoides* ethanolic extract; LgEE: *Lippia gracilis* ethanolic extract.

**Table 5:** Bacterial origin and antibiotic resistance profile.

Bacterial Strain	Origin	Resistance Profile
*Staphylococcus aureus* Sa 358	Surgical Wound	AMK, BTN, CPN, GEN, NEO, NET, OXA, PRM, SISO, TOB.
*Escherichia coli* Ec 27	Surgical Wound	AMK, AMP, AMX, AZM, CAZ, CEC, CEF, CHL, CIP, CPN, IPM, KAN, SMX, TET, TOB.

AMK: amikacin; AMP: ampicillin; AMX: amoxicillin; AZM: azithromycin; BTN: butirosin; CAZ: ceftazinidime; CEC: cefaclor; CEF: cephalothin; CHL: chloramphenicol; CIP: ciprofloxacin; CPN: cephalexin; GEN: gentamicin; IPM: imipenem; KAN: kanamycin; NEO: neomycin; NET: netilmicin; OXA: oxacillin; PRM: paramomycin; SISO: sisomicin; SMX: sulfamethoxazole; TET: tetracycline; TOB: tobramycin. Adapted from Sobral et al. [91].

## Data Availability

The data will be available after a reasonable request to the corresponding authors.
